# Dr. Waterman Pardoned

**Published:** 1846-07

**Authors:** 


*Dr. Waterman pardoned.*—Dr Waterman whose sentence to the State prison for three years for being accessory to the disinterment of subjects for dissection, was noticed in a previous number of our journal, has been pardoned by Gov. Wright. We learn that during his confinement his services were very acceptable in the hospital department.

